# Linkage of the Medical Education Partnership Initiative (MEPI) and the Nursing Education Partnership Initiative (NEPI) to the African Forum for Research and Education in Health (AFREhealth)

**DOI:** 10.29024/aogh.2

**Published:** 2018-04-30

**Authors:** Nelson K. Sewankambo, Peter Donkor

**Affiliations:** 1 Member AFREhealth Council and PI of Medical Education for Equitable Services to All Ugandans (MESAU); 2 Chair of the AFREhealth Council and PI of the MEPI Emergency Medicine Collaborative, KNUST, GH

## Abstract

The US Government funded Medical Education Partnership Initiative (MEPI) and the Nursing Education Partnership Initiative (NEPI) were large undertakings that introduced major creative changes and innovations in the approach to education of health professions and research in the participating medical and nursing institutions. The African-led MEPI Principal Investigators’ (PI) Council which provided strong leadership resolved not to lose momentum but to scale up beyond the MEPI and NEPI institutions and continue with shared learning and the transformative changes that had emerged. The Council started a multi-professional platform which would facilitate and support inter-professional collaboration as the best way forward in the post-MEPI and NEPI period. The African Forum for Research and Education in Africa (AFREhealth) was started and will have to show the value addition it brings and how it will ensure its own sustainability while strengthening South to South partnerships.

This supplement has been put together by the African Forum for Research and Education in Health (AFREhealth)[1]. There are 21 papers contributed by professionals who were engaged, between 2010 to 2017, in the medical education partnership initiative (MEPI) and the nursing education partnership initiative (NEPI) activities and those who were not [2,3]. Two common characteristics of these authors are either being members of AFREhealth and/or having a shared interest in the aspirations of the organisation and hence a desire to support AFREhealth and contribute to developing the African continent. There is a lot of goodwill towards AFREhealth from within and outside Africa. This is exemplified by the fact that for this supplement, 46 potential reviewers were approached from Africa, Europe and North America, and all except two returned the reviews within a of three weeks. One potential reviewer did not respond to our request, while one other informed us of his regret within three days that he was unable to review the paper because he was already overcommitted during the period the review was required. The authors themselves responded to the reviewers’ and editors’ comments within two to three weeks. We were extremely impressed by this level of support and commitment from both the authors and reviewers. AFREhealth should work tirelessly to tap into and make maximum use of this goodwill. What is AFREhealth and what are its origins?

In contrast to how Africa has often been portrayed in the media and scientific literature, AFREhealth is firmly rooted in the positive thinking and optimism that the African continent holds a lot of promises for the future. In a recent report, “The path to longer and healthier lives for all Africans by 2030: The Lancet Commission on the future of health in sub-Saharan Africa,” the Commissioners’ vision is that Africans should expect the same opportunities for health by 2030 as all other people [4]. The Commission also observed that to achieve these results it should not be business as usual with regards to policies and programs. A notable development in the 21st century is that Africa’s economy is rising. However, to ensure sustainable development there must be significant, steady improvements in the health outcomes of the population driven in part by appropriate innovations in education of the health workforce, research and service delivery. One of Africa’s challenges is how to produce well-qualified health workers that are fit-for-purpose, available in adequate numbers, retained and well distributed in both rural and urban environments so as to ensure universal health coverage and to overcome inequities in access to quality services. The US government funded MEPI and NEPI projects have both demonstrated that innovations by Africans are indeed possible and that there are many opportunities including feasibility for South-South collaboration and joint learning in Africa [2,3]. These two initiatives are the largest individual projects ever initiated are aimed at transforming medical and nursing education on the continent. They have transformed the minds of policy makers in government departments, educational institutions both public and private, health professional educators and their students and some funders and the general public. The expected outcomes are improvements of health outcomes, including attainment of an AIDS-free generation, supported by evidence-based interventions, a strong research enterprise addressing health priorities in Africa, and mobilization of the needed vital resources. The generous funding from the US government has come to an end, and yet a lot remains to be done. How can the momentum gained by MEPI and NEPI be sustained? This issue has preoccupied the minds of many Africans who have as a result looked for potential solutions. AFREhealth was started to address this challenge.

The AFREhealth was launched in August 2016 during the annual MEPI symposium held in Nairobi, Kenya, with a vision to build inter-professional partnerships across professions for education, research and service delivery. It is envisaged that an inter-professional approach will go a long way in ultimately improving health services and health status of our populations. The activities under MEPI and NEPI slowly evolved into what has become a social movement. There was a need to build on the legacy of these two initiatives that became brand names in a period of less than eight years since their inception. Unlike many regional or continental health initiatives in Africa, AFREhealth is built on a determination by African health professionals to take charge of the continent’s future through mechanisms that are African initiated, African owned and African led to determine priorities (in education, research and service delivery) for promoting the highest level of collaboration across the entire continent. AFREhealth is open to health professions education and research institutions irrespective of whether they were members in MEPI or NEPI or not.

Partnerships and collaboration across professions (South-South, South-North and North-South) will be key elements of the Forum’s activities moving forward. We must bring south-south collaboration to a new level and strengthen south-south and north south partnerships. We will also engage with Africans in the diaspora and well-wishers overseas. The Forum’s niche is on inter-professional approaches and we will work with existing and emerging health professional networks and organisations that share common goals, without discrimination. The Forum has to show its added value to these entities, minimize competition and create synergy. It aims to partner closely with national governments and other stakeholders in both public and private sector.

A strong and committed African leadership with a clear vision and active engagement of all stakeholders will be vital in ensuring success. AFREhealth has a dynamic leadership platform composed of a mix of African health professionals committed to advance, the state of health on the continent. The governance model of having a Principal Investigators’ (PI) Council as the top policy organ served MEPI well and has been adopted but with an expanded membership. This multi-professional membership council aligns well with the vision of the forum. AFREhealth has already demonstrated its organising potential and convening power by successfully holding a symposium in Accra, Ghana (Aug 1–3, 2017). The annual symposium is a platform for sharing best practices and learning about new innovations and research findings relevant or applicable to African environments. The AFREhealth Council pulled this off successfully and we look forward to many such meetings to be held in a rotating manner from one country to another. The success of this meeting was a surprise to those who believed that in the absence of MEPI and NEPI funding the meeting would not take place.

Working together through AFREhealth is likely to give a louder voice to the academia in African academic institutions and become more active players in advising their national governments on policies for human resource development and deployment. Countries would become more self-reliant instead of depending on consultants from elsewhere in high income countries. The forum will therefore have to be creative in how it actively engages with policy makers. An obvious issue for such engagement is providing evidence and convincing national governments on the need to invest more financial resources to improve quality and quantity of health workforce training and education, their appropriate deployment and support. AFREhealth itself will need funding support if it is to succeed and be able to sustain its activities.

## References

[1] Omaswa F, Kiguli-Malwadde E, Donkor P, et al., Medical Education Partnership Initiative gives birth to AFREhealth. Lancet Global Health. 2017; 5(10): 965–966. DOI: 10.1016/S2214-109X(17)30329-7 PMC5712669 28911758

[2] Omaswa F, Kiguli-Malwadde E, Donkor P, et al. The Medical Education Partnership Initiative (MEPI): Innovations and lessons for health professions training and research in Africa. Supplement AOGH; 2017. 10.29024/aogh.8 PMC6586907 30873813

[3] Michaels-Strasser S, Smith J, Khanyola J, Sutton R, Price T and El Sadr W. Strengthening the Quality and Quantity of the Nursing and Midwifery Workforce: A high-level report on eight years of the NEPI Project. AOGH Supplement; 2017. 10.29024/aogh.6 PMC6748304 30873797

[4] Agyepong IA, Sewankambo N, Binagwaho A, et al. The path to longer and healthier lives for all Africans by 2030: The Lancet Commission on the future of health in sub-Saharan Africa. Lancet; 2017 DOI: 10.1016/S0140-6736(17)31509-X 28917958

